# Ultrasonographic Diagnosis of Urachal Anomalies in Cats and Dogs: Retrospective Study of 98 Cases (2009–2019)

**DOI:** 10.3390/vetsci7030084

**Published:** 2020-07-02

**Authors:** Francesca Perondi, Caterina Puccinelli, Ilaria Lippi, Daniele Della Santa, Michelangelo Benvenuti, Tommaso Mannucci, Simonetta Citi

**Affiliations:** 1 Department of Veterinary Sciences, University of Pisa, Via Livornese Lato Monte, 56121 Pisa, Italy; f.perondi87@gmail.com (F.P.); caterina.puccinelli@phd.unipi.it (C.P.); tommy.mannucci@gmail.com (T.M.); simonetta.citi@unipi.it (S.C.); 2 Vet Hospital H24, 50142 Firenze, Italy; danieledellasanta@yahoo.it (D.D.S.); miche-sportster.hd@libero.it (M.B.)

**Keywords:** dog, cat, urachal anomalies, vesicourachal diverticula, ultrasound

## Abstract

This retrospective study investigated the prevalence of different urachal anomalies (UA) in cats (*n* = 60) and dogs (*n* = 38) and their association with clinical symptoms and urinalysis alterations. Among UA, the vesicourachal diverticulum was the most prevalent UA diagnosed in both cats (96.7%) and dogs (89.5%): the intramural vesicourachal diverticulum was diagnosed in 76.7% of cats and 71.1% of dogs, followed by extramural vesicourachal diverticulum (20.0% and 18.4% respectively). In both cats and dogs, bladder wall diffuse or regional thickening was the most prevalent alteration. The most common alterations of the urinary bladder content were urolithiasis sediment in cats (33.3%) and in dogs (31.6%). Dogs with UA were more often asymptomatic (*p* = 0.01). No difference was found in cats. Stranguria, hematuria, and urethral obstruction were the most frequently reported clinical signs, while hematuria and leukocyturia were the most prevalent abnormalities at urinalysis. In conclusion, our study confirmed UA as uncommon, and often incidental findings, with a high prevalence of animals without clinical signs.

## 1. Introduction

The urachus is a fetal connection allowing urine to pass between the developing urinary bladder and the placenta [1,2,3]. Urachal anomalies (UA) result from failure of the urachus to undergo complete atrophy by the time of birth: at this time it should be nonfunctional and typically look like a fibrous connective tissue remnant connecting the bladder vertex with the umbilicus [4,5]. The factors responsible for incomplete closure and atrophy of the urachus have not yet been defined [5]. UA are relatively uncommon congenital diseases of the lower urinary tract of dogs and cats [3,4,6].

**Figure 1:**
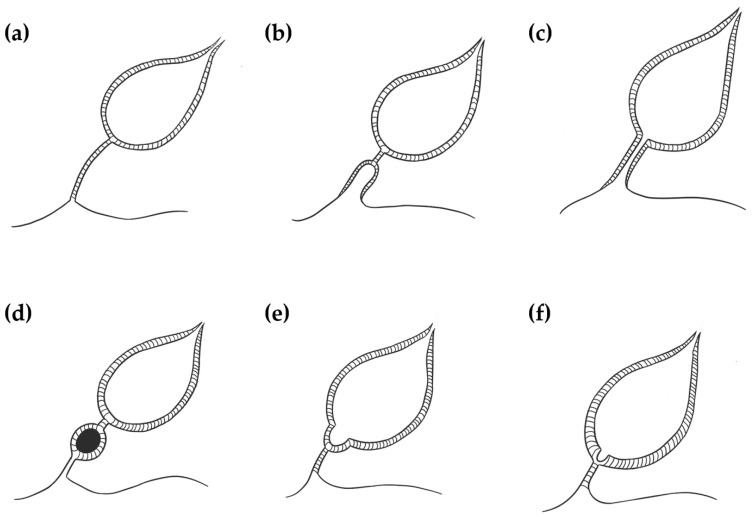
Illustration of the different types of urachal anomalies. (**a**) Normal bladder with a completely atrophied urachus; (**b**) urachal sinus: the distal part of the urachus remains patent and communicates with the umbilicus; (**c**) patent urachus: the urachal canal remains patent and connects the bladder to the umbilicus; (**d**) urachal cyst: a focal segment of the patent urachus remains persistent; (**e**) vesicourachal diverticulum (extramural): the portion of the urachus located at the bladder vertex fails to close, resulting in a blind diverticulum protruding beyond the serosal surface of the bladder; (**f**) vesicourachal diverticulum (intramural): in this case the blind diverticulum is limited to the thickness of the bladder wall.

Four different UA have been described in dogs and cats: patent urachus, urachal cysts, vesicourachal diverticula, and urachal sinus (Figure 1) [3,4,6].

A patent (or persistent) urachus occurs when the urachal canal remains functionally patent between the bladder and the umbilicus. It is characterized by inappropriate urine loss through the umbilicus and can also be associated with omphalitis and urinary tract infection [3,4]. Urachal cysts develop when the umbilical and bladder ends of the urachus are obliterated, but a focal segment of a patent urachus remains persistent [4,7]. A vesicourachal diverticulum occurs when a portion of the urachus located at the bladder vertex fails to close, resulting in a blind diverticulum that protrudes from the bladder apex [3,4]. Vesicourachal diverticula may be classified as intramural or extramural based on their anatomical extent [4,8]. A urachal sinus occurs when the distal part of the urachus remains patent and communicates with the umbilicus. This is often asymptomatic and rarely recognized [4].

UA are often under-diagnosed conditions that may be found incidentally at imaging. Associated clinical signs may be attributed to urinary tract infection (UTI) and may be indistinguishable from other acquired causes of lower urinary tract diseases (LUTD) or affected patients can be completely asymptomatic [5,7,9].

In human medicine, the reported incidence of urachal anomalies is relatively low, approximately one in 5000 for adults (less among infants). The most common type of urachal anomaly reported is a patent urachus (47%), followed by urachal cyst (30%), sinus (18%), and, least commonly, vesicourachal diverticulum (3%) [7]. In veterinary medicine, the prevalence is unknown, because there are only few studies and reviews [3,8,10] or case reports [11,12,13].

Although UA can be diagnosed by different imaging techniques (ultrasonography, contrast cystography, or cystoscopy) ultrasonography is the most commonly used, because of its widespread availability and absence of ionizing radiation exposure [3,6,7,14,15,16].

The purposes of the present retrospective study are to determine (1) the prevalence of the different types of urachal anomalies in cats and dogs (2) the association of UA with urinary clinical signs and urinalysis abnormalities.

## 2. Materials and Methods

Medical records (*n* = 28,174) of the Veterinary Teaching Hospital of the Department of Veterinary Science (University of 61 Pisa, Italy) and Veterinary Clinical Practice “Vet Hospital H24” (Florence, Italy) between July 2009 and July 2019 were retrospectively reviewed for client-owned dogs and cats with an ultrasonographic diagnosis of urachal anomalies.

For each case with diagnosis of UA, data regarding signalment, history, physical examination and urological clinical findings (hematuria, pollakiuria, stranguria or urinary incontinence), abdominal ultrasound, and urinalysis findings (bacteriuria, leukocyturia, hematuria and crystalluria) were collected from the medical record when available. Patients were considered symptomatic in case at least one of the following signs of lower urinary tract disease was present: hematuria, pollakiuria, stranguria, urinary incontinence. In case none of the symptoms was present, patients were defined as asymptomatic. All cases with uncertain ultrasonographic diagnosis have been excluded.

Ultrasonography was performed using a Toshiba Aplio 400 (Canon Medical Systems Europe B.V., Zoetermeer, The Netherlands), and General Electrics Logiq E, Logiq E R6 and logiq E R7 equipped with 3,5-10 MHz microconvex (8C-RS), 4,5-13 MHz (12L) and 6,7-18 (L8-18i-RS) linear probes; patients were scanned in lateral recumbency.

Ultrasonographic records, images, and videos were reviewed for each patient with urachal anomalies. Data regarding ultrasound appearance of the wall and the content of the urinary bladder were also recorded for each patient. Urachal anomalies were then classified as follows: patent urachus, defined as a fluid-filled tubular structure, extending from the cranioventral bladder wall to the umbilicus; urachal cyst, defined as a thin-walled, fluid-filled structure, cranial to the bladder; vesicourachal diverticulum defined as a fluid filled structure extending from the bladder cranioventrally as a convex out-pouching of the lumen, subclassified as intramural (limited to the thickness of the bladder wall) or extramural (protruding beyond the serosal surface of the bladder) [3,17,18,19].

Moreover, the presence of other urinary bladder anomalies was also recorded: bladder anomalies were then divided into content anomalies and wall anomalies. Content anomalies were classified as the presence of calculi (intraluminal mobile structures, of variable size, number and shape, with a hyperechoic interface and forming distal shadow); sediment (intraluminal material of variable echogenicity, which layers in the dependent portion of the bladder and suspends with agitation of the urinary bladder); hyperechoic bands (multiple intraluminal suspended hyperechoic strips, compatible with necrotic, fibrinous and haemorrhagic material); and blood clot (free intraluminal nonshadowing structure, of variable size, shape, and echogenicity) [17,18,20]. Wall anomalies were defined as the presence of wall thickening and were classified as diffuse or regional thickening and focal thickening (wall mass extending into the bladder lumen) [17,18].

Urine samples were collected and analyzed within 12 h from collection (IDEXX VetLab UA Analyzer and Idexx UA Strips, Idexx, Milan, Italy). Urinary sediment reports were reviewed to check for the presence of bacteriuria, leukocyturia, hematuria, and crystalluria.

The D’Agostino and Pearson normality test was used to test data for normality using Graphpad Prism 4 (Graph Pad, San Diego, CA, USA). Chi-squared test was used to compare the prevalence of asymptomatic and symptomatic dogs and cats in relation to different urachal anomalies.

## 3. Results

During the study period, 28,174 abdominal ultrasounds (6450 cats and 21,724 dogs) were performed for different clinical indications, resulting in an ultrasonographic diagnosis of UA in 98 cases (60 cats and 38 dogs). The prevalence of UA was 0.93% in cats and 0.18% in dogs.

Most of the cats were of mixed lineage (*n* = 56, 93.3%), four cats were reported as pure breed represented breed include Persian Cats (2 cats, 3.3%), Ragdoll and Norwegian Forest Cat (one cat each, 1.6%). Forty-six cats (76.7%) were male (6/46 intact male and 40/46 were castrated male) and 14 cats (23.3%) were female (3/14 were intact female and 11/14 spayed female). The median age was 5 years (ranging from 4 months to 15 years), 95% of cats were >1 years. Dogs were represented by the following breeds: mixed breed (12 dogs, 31.5%), Boxer (6 dogs, 15.8%), Golden Retriever (4 dogs, 10.5%), Newfoundlander, English Cocker Spaniel, Labrador Retriever (2 dogs each, 5.3%) and Beagle, German Shepherd dog, Dalmatian, Papillon, American Staffordshire Terrier, Dogue de Bordeaux, ShihTzu, Jack Russell Terrier, Rottweiler, and Poodle (one dog each, 2.6%). Thirty-one dogs (81.6%) were male (25/31 were castrated male and 6/31 were intact male) and seven (18.4%) were intact female. The median age was 8.5 years (ranging from 2 months to 15 years) and 18% were <1 years old.

Vesicourachal diverticulum was the most prevalent UA diagnosed in this study in both cats (96.7%) and dogs (89.5%) (Table 1).

**Figure 2:**
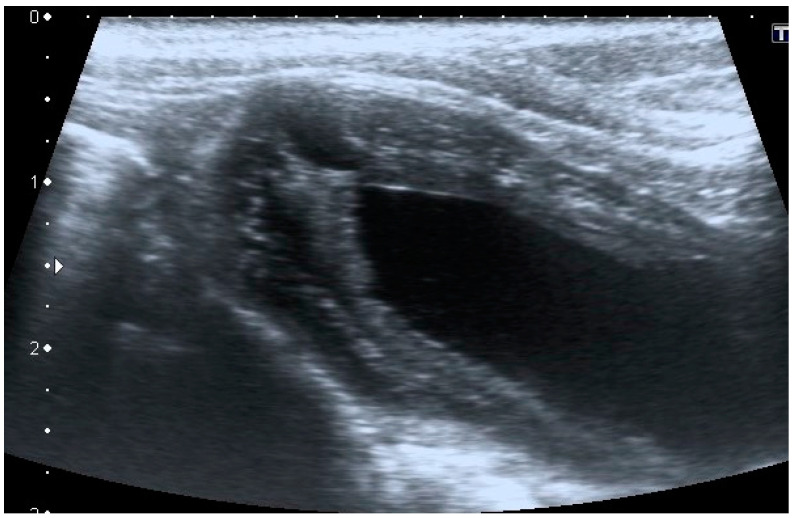
Longitudinal ultrasound image of the urinary bladder in a dog, showing the presence of a small (<1 cm diameter), well-defined, fluid-filled, anechoic structure in the cranioventral bladder wall, consistent with intramural vesicourachal diverticulum.

Intramural vesicourachal diverticulum was the most prevalent UA diagnosed in both cats (76.7%), and dogs (71.1%). These varied in size, from thin anechogenic lines perpendicular to the wall, in the ventro-cranial portion of the bladder, to formations of greater width, up to 0.4–0.5 cm. Its content was variable, from anechoic to slightly corpuscular, in relation to the bladder corpuscularity (Figure 2).

**Figure 3:**
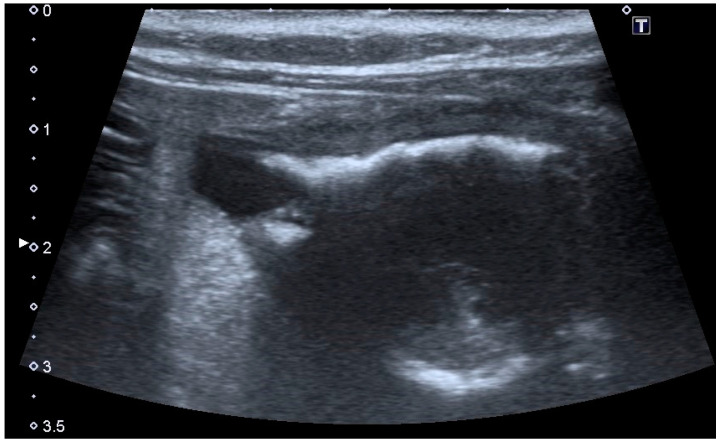
Longitudinal ultrasound image of the urinary bladder in a cat, showing the presence of a small (<1 cm diameter), well-defined, fluid-filled, anechoic structure, protruding beyond the serosal surface of the cranioventral bladder wall, consistent with extramural vesicourachal diverticulum. The bladder presents a moderate, diffuse, thickening of the wall, with an irregular mucosal surface. A moderate amount of echogenic sediment is present in the lumen. Shadowing hyperechoic material adhered to the ventral bladder wall mucosa is also observed, which may represent congealed mineralized sediment.

Extramural vesicourachal diverticulum was diagnosed in 20.0% of cats and 18.4% of dogs. Four out of 19 were of large dimensions and extended 1 to 2 cm cranially to the serosa of the urinary bladder apex; 3/19 appeared 0.5–1 cm in diameter, while 12/19 were smaller than 0.5 cm. The wall appeared as a thin hyperechoic line and the content was similar to that of the bladder (Figure 3).

**Figure 4:**
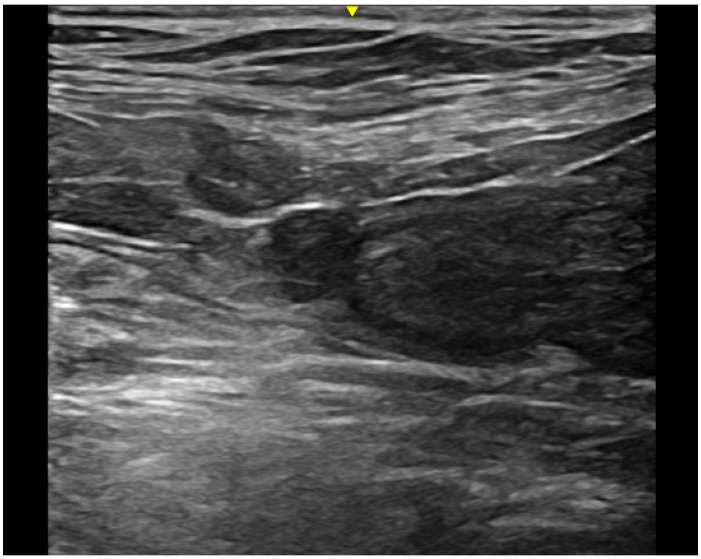
Longitudinal ultrasound image of the cranioventral part of the urinary bladder in a cat, showing the presence of a small (<1 cm diameter), well-defined, round, thin-walled, anechoic formation, cranial to the bladder apex, consistent with urachal cyst. The urinary bladder is empty.

Urachal cyst: only five patients (two cats and three dogs) had this UA. Urachal cysts appeared as round structures in continuity with the bladder wall, with thin and hyperechoic walls and regular margins. It was of different size, from 0.2 to 0.5 cm and the content was always anechoic (Figure 4).

**Figure 5:**
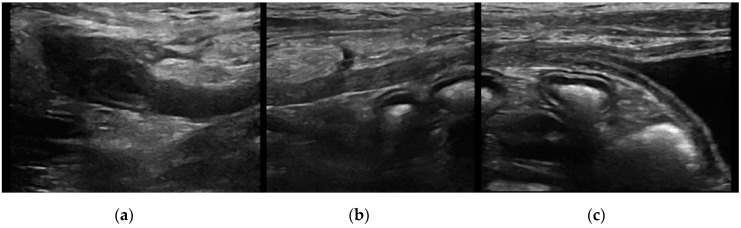
Longitudinal ultrasound images of a patent urachus in a dog. A tubular structure (≤5 mm diameter) with a small amount of anechoic content extends from the cranio-ventral bladder wall to the umbilical region. Mild reactivity of the peritoneal fat and a small quantity of peritoneal fluid are present in proximity of the urachus. (**a**) Terminal urachus part reaching the umbilical region; (**b**) intermediate urachus part; (**c**) urachus part in continuity with the bladder apex.

Patent urachus was present in only one mixed-breed dog of 6 months of age. The urachal presented a tubular structure and anechoic content. The formation began from the bladder and ended at the umbilicus (Figure 5).

No urachal sinus was diagnosed during the period of the retrospective study.

Twenty-three out of 60 (38.3%) cats did not present bladder content anomalies at ultrasound evaluation, and 28/60 (46.6%) cats did not present any bladder wall anomaly; 20/60 (33.3%) had sediment and 30/60 (50%) reported diffuse wall thickening (Table 2).

Eighteen out of 38 dogs (47.4%) did not have any other bladder anomalies at ultrasound evaluation. Twenty out of 38 dogs (52.6%) had other alterations: calculi were the most prevalent content anomaly (13/38, 31.6%), and diffuse thickening was the most prevalent wall anomaly (16/38, 42.1%) (Table 2).

On history, 23/60 cats (38.3%) and 22/38 dogs (57.9%) had no urinary clinical signs (Table 3). In particular, in cats, no statistically significant difference in the percentage of patients with or without clinical signs was found (*p* = 0.56) for both intramural and extramural vesicourachal diverticulum, although a non-significant trend of higher percentage of patients with clinical signs was noticed. While in dogs the percentage of animals with no clinical signs was significantly higher (*p* = 0.01) than the percentage of animals with clinical signs, for both intramural and extramural vesicourachal diverticulum.

For intramural vesicourachal diverticulum, stranguria was the most frequent alteration (28.8% and 21.4% for cats and dogs, respectively), followed by pollakiuria (17.7% for cats and 17.8% for dogs), while 37.7% of cats and 53.6% of dogs were without clinical signs.

Thirteen out of 37 cats (35.0%) and 6/16 dogs (37.5%) with urinary clinical signs were presented in emergency for urethral obstruction.

Fifty-two out of 98 (36 cats and 16 dogs) had urinalysis available for review. Leukocyturia and hematuria were the most prevalent urinalysis anomalies in both cats (24/36, 66.6% and 30/36, 83.3%, respectively) and dogs (12/16, 75.0% and 10/16, 62.5%, respectively). Crystalluria was present in 31.2% of dogs and in 52.7% of cats, while bacteriuria was present, respectively, in 25.0% and 27.8% of dogs and cats. In particular for intramural vesicourachal diverticulum, leukocyturia and hematuria were present, respectively, in 83.3% and 58.3% of dogs, and in 63.3% and 86.6% of cats.

In cats, crystalluria was present in 53.3% of cats with intramural vesicourachal diverticula, 25.0% of cats with extramural vesicourachal diverticula and in one cat with urachal cyst and patent urachus. Crystalluria was present in 41.6% of dogs with intramural vesicourachal diverticulum, while it was absent in the remaining UA. In cats, crystalluria was due to struvite in 13/16 cats (81.3%) and to calcium oxalate in 3/16 cats (18.7%). In dogs, crystalluria was due to struvite in 3/5 dogs (60.0%), to calcium oxalate in 1/5 dogs (20.0%), and to cystine in 1/5 dogs (20.0%).

## 4. Discussion

Urachal anomalies are uncommon disorders of the urinary bladder, and often incidental findings in veterinary medicine [3,4,5,8]. The real incidence is yet unknown because there are only a few case reports [11,12,13,21] or reviews [3,4,5,10] concerning this topic. Similar to veterinary medicine, the incidence of urachal anomalies in human medicine is relatively low [7,22], with the majority of cases showing no clinical signs, and being diagnosed incidentally [7]. In particular, urachal anomalies are more frequently diagnosed in children, where an incidence of 1 case on 7.610 for patent urachus and 1 case on 5.000 of urachal cyst was reported [22].

**Figure 6:**
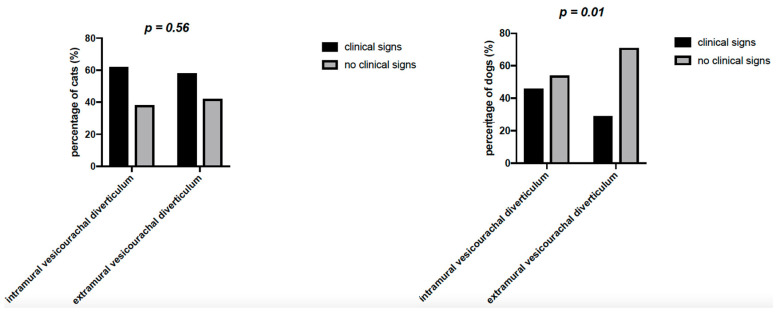
Chi squared test of the percentage of cats and dogs with clinical signs and without clinical signs, for both intramural and extramural vesicourachal diverticulum.

In our study, the prevalence of UA was 0.93% in cats and 0.18% in dogs, similar to what is reported in humans. Interestingly, only 5% of cats and 18% of dogs diagnosed with UA were < 1 year old. In our study, a median age of 5 years (4 months–15 years) was reported for cats, and a median age of 8.5 years (2 months–15 years) for dogs. Median age at diagnosis seems higher in veterinary patients compared to humans. This finding is in agreement with previous studies in veterinary medicine, in which mean age at diagnosis was far above 1 year old [4,8]. In a study of 24 clinically normal cats with radiographic evidence of an intramural vesicourachal diverticulum, a mean age of 2.5 years was reported, while in another study of 149 symptomatic cats with diverticula, a mean age of 3.7 ± 2.7 years was reported [4]. Mean age at diagnosis was even higher in dogs, where Groesslinger and colleagues reported a mean age of 10.4 ± 4.4 years, with only 8% of dogs < 1 years old [8]. The authors hypothesize that the increased median age at diagnosis of UA in our cohort is related to the high prevalence of asymptomatic patients. UA was often an incidental finding in patients requiring ultrasonography for reasons other than urinary tract disease. In our study the majority of dogs (57.9%) were asymptomatic. This finding was in agreement with a previous study [8], in which the diagnosis of UA was not associated with clinical signs of UTI in 34% of dogs [8]. In the cats of our cohort the prevalence of asymptomatic subjects was lower than that found in dogs (38.3%). In particular, while the number of dogs with no clinical signs was significantly higher than the number of dogs with clinical signs, for both intramural and extramural diverticulum, this difference was not statistically evident in cats (Figure 6). Unpublished clinical experience showed that clinical signs of LUTD are a more frequent reason for ultrasonography in feline, rather than in canine patients. As a consequence, the number of cats requiring ultrasonography, with signs of LUTD may be relatively higher than in dogs. Although it is possible that the diagnosis of UA is more frequently associated with clinical signs of LUTD in cats than in dogs, this finding is difficult to interpret due to the fact that previous data in cats are limited to few case reports [5,11].

In symptomatic animals, hematuria, dysuria, pollakiuria, and urethral obstruction were the most commonly reported clinical signs associated with UA in the current literature [4,5,12]. In our cohort of patients, the most prevalent clinical symptoms associated with UA were stranguria and pollakiuria, particularly in dogs and cats with intramural vesicourachal diverticula. Interestingly, urethral obstruction was present in 35% of cats and in 37.5% of dogs with urinary symptoms. It is plausible that in patients with urethral obstruction the diagnosis of UA may be facilitated by a condition of bladder overfill. This finding seems in agreement with the results of a previous study of Osborne and colleagues, in which urethral obstruction was reported in the 54.5% of cats with vesicourachal diverticula [4]. The largest diverticula were found in cats with complete urethral outflow obstruction, supposedly due to the increased intraluminal pressure in the bladder lumen [4].

Unfortunately, urinalysis was only available in a limited number of dogs and cats. Interestingly, the majority of dogs and cats for which urinalysis was available had signs of inflammation, such as hematuria, leukocyturia and crystals. However, it is to be noted that dogs and cats with urinalysis were those patients for which abdominal ultrasonography was performed to investigate urologic clinical signs. Therefore, the prevalence of urinary signs of inflammation may be overestimated.

**Figure 7:**
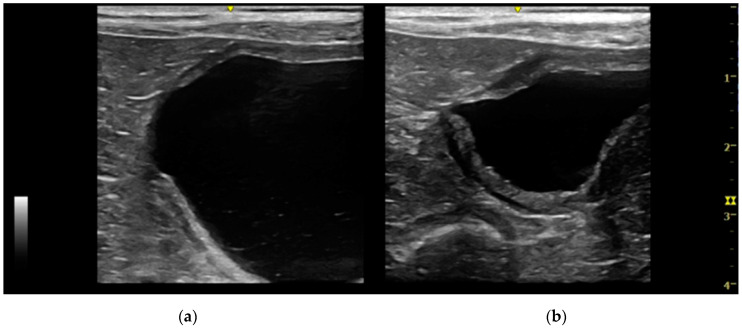
Two longitudinal ultrasound images of the urinary bladder of a dog with an intramural vesicourachal diverticulum. The images show two different volumes of urine into the bladder lumen. (**a)** Overly distended: the diverticulum appears as a not very visible focal depression in the cranial-ventral aspect of the bladder, with thinning of the correspondent bladder wall; (**b)** ddequately distended: the diverticulum appears as an evident, focal, evagination in the cranio-ventral aspect of the bladder.

Historically, the diagnosis of UA in veterinary medicine was performed by the use of different imaging techniques, such as ultrasonography, contrast cystography, and cystoscopy [3,14,23]. In previous studies, contrast cystography was used to diagnose UA [8,12]. According to Osborne and colleagues, positive antegrade cystourethrography and retrograde positive contrast urethrocystography were considered the procedures of choice for the diagnosis of vesicourachal diverticula [4]. In the present study the diagnosis of UA was based on abdominal ultrasonography only. Abdominal ultrasound has the advantages to be a routinely available and non-invasive imaging technique. Similarly, in human medicine, abdominal ultrasound is considered a fast and readily available technique to diagnose UA in absence of the risks connected to radiation exposure [7]. However, we should consider that the diagnostic power of abdominal ultrasonography in identifying UA may be reduced by a condition of inadequate bladder filling. In one of the patients of our study, the diagnosis of intramural vesicourachal diverticulum was made at the second ultrasonography, when the bladder appeared less filled (Figure 7). Therefore, we cannot exclude that small diverticula may be undiagnosed by the use of ultrasonography, in case of inadequate bladder filling.

In our cohort, the vesicourachal diverticulum was the most frequently diagnosed UA, with a prevalence of 96.7% in cats and 89.5% in dogs. In particular, the prevalence of intramural vesicourachal diverticulum was 76.7% in cats and 71.1% in dogs. This was followed by extramural vesicourachal diverticulum (18.4% and 20% in dogs and cats, respectively). In veterinary medicine, vesicourachal diverticulum (both microscopic and macroscopic) seemed to be the most frequent UA in dogs and cats [4,8]. For feline patients, one study [24] reported a prevalence of 40% of microscopic diverticula, while another study [4] reported a prevalence of vesicourachal diverticulum of 22% in cats with urinary symptoms. For canine patients, one study reported a prevalence of vesicourachal diverticulum of 34% [8]. In veterinary medicine, there is only one study reporting a different prevalence between intramural (19/33, 57.6%) and extramural diverticula (14/33, 42.4%) in 33 cats with bladder diverticula [4]. In another study of 50 dogs, only 1/17 vesicourachal diverticula was classified as extramural, confirming also in the dog, the lower prevalence of extramural diverticula compared to intramural [8]. Veterinary patients seem to differ from human patients, for whom patent urachus was the most prevalent UA (47%), followed by urachal cyst (30%), and vesicourachal diverticulum (3%) [7]. In our study the prevalence of urachal cysts and patent urachus was very low in both cats and dogs. This finding was in agreement with what was previously described in both case reports [11,21] and reviews [4,5]. The urachal cysts may be under-diagnosed because they are located along the course of the urachus, but they are not necessarily associated and/or adjacent to the bladder, and for example they could be misinterpreted as mesenteric cysts.

No urachal sinus was diagnoses during the period of the retrospective study. One possible explanation could be that the urachal sinus is often associated with non-specific symptoms and is largely asymptomatic unless it develops a complication (most commonly infection). [4,25,26] Another possible explanation could be its anatomical location; indeed, it is not located near the urinary bladder and if asymptomatic it may not be observed during an abdominal ultrasound examination.

In our cohort of patients, UA was frequently associated with other ultrasound alterations of the bladder in terms of content and/or wall anomalies. In both dogs and cats, diffuse or regional thickening was the most prevalent bladder wall alteration. The most common alterations of the urinary bladder content were urolithiasis in dogs and sediment in cats. Similar findings were reported by the study of Osborne and Colleagues [4], in which the most prevalent abnormalities in patients with UA were thickened bladder wall (75%), irregular mucosa (44%), calculi and sediment (33%). These findings, together with unpublished clinical experiences may suggest a potential role of UA in promoting urolithiasis, and/or inflammatory changes of the bladder mucosa. UA may host bacteria, or protein and mineral debris, which may be difficult to clear by the physiologic urine flow. Some studies also reported that macroscopic diverticula may develop from microscopic diverticula, with the concomitant presence of disorders of the lower urinary tract, such as bacterial infections, urolithiasis, urethral plugs, sediment, or idiopathic cystitis. However, the exact relationship between vesicourachal diverticula and other disorders of the lower urinary tract is still unknown [4,27,28,29,30].

This study has some limitations. Due to the retrospective nature of the study, cases with incomplete or uncertain ultrasound diagnosis were excluded from the study. Therefore, it is possible that the exact prevalence of UA may be slightly affected. Data regarding urinalysis were available only for a limited number of subjects. In some cases, urinalysis was not performed as the patients were referred to the hospital only for the ultrasonographic examination, in other cases urinalysis was not included in the initial diagnostic plan. In both cases, the lack of urinalysis might affect our results, in terms of prevalence of signs of urinary inflammation. Moreover, as in the majority of our cases UA were incidental findings, no urine culture was available. The lack of urine culture did not allow us to make any speculation on the prevalence of UTI in these patients. Finally, as abdominal ultrasonography was the only imaging technique used to identify UA, it is possible that some patients with UA were undiagnosed, especially if urinary bladder was inadequately replete, and/or in the case of small diverticula.

## 5. Conclusions

In conclusion, our study confirmed urachal anomalies as uncommon and often incidental findings, with a high number/proportion of patients without urinary clinical signs. When present, clinical signs were mostly characterized by hematuria, stranguria, and/or urethral obstruction. Different from human medicine, UA were diagnosed in dogs and cats of any age, with a lower prevalence in patients < 1 year old. Among different UA, intramural and extramural vesicourachal diverticula were the most prevalent in both canine and feline populations, often associated with other ultrasound signs of bladder inflammation. Abdominal ultrasound is a routinely available and non-invasive imaging technique to investigate UA. However, additional imaging techniques (such as contrast cystography) or repeated abdominal ultrasounds should be considered in the case of inadequate bladder filling and/or in cases of patients with clinical signs where a specific management could be important.

## Figures and Tables

**Table 1:** Prevalence of the different type of Urachal Anomalies.

Urachal Anomalies	Cats (*n* = 60)	Dogs (*n* = 38)
Vesicourachal diverticulum	58 (96.7%)	34 (89.5%)
Intramural vesicourachal diverticulum	46/60 (76.7%)	27/38 (71.1%)
Extramural vesicourachal diverticulum	12/60 (20.0%)	7/38 (18.4%)
Urachal cysts	2 /60 (3.3%)	3/38 (7.9%)
Patent urachus	0	1/38 (2.6%)
Urachal sinus	0	0

**Table 2:** Prevalence of urinary bladder content and wall anomalies in all patients included with different type of urachal anomalies.

Urachal Anomalies	Species	Urinary Bladder Content Anomalies	*n*	Urinary Bladder Wall Anomalies	*n*
Intramural vesicourachal diverticulum	Cats	Calculi Sediment Blood clot Hyperechoic bands No anomalies	8/45 15/45 1/45 3/45 18/45	Diffuse or regional thickening Focal thickening No anomalies	25/45 1/45 19/45
	Dogs	Calculi Sediment No anomalies	10/28 8/28 10/28	Diffuse or regional thickening Focal thickening No anomalies	14/28 4/28 10/28
Extramural vesicourachal diverticulum	Cats	Calculi Sediment No anomalies	5/12 3/12 4/12	Diffuse or regional thickening No anomalies	4/12 8/12
	Dogs	Calculi No anomalies	1/7 6/7	Diffuse or regional thickening No anomalies	1/7 6/7
Urachal cysts	Cats	Sediment	2/2	Diffuse or regional thickening Focal thickening	1/2 1/2
	Dogs	Calculi No anomalies	1/3 2/3	Diffuse or regional thickening No anomalies	1/3 2/3
Patent urachus	Dogs	No anomalies	1/1	No anomalies	1/1

**Table 3:** Prevalence of clinical urinary findings in all patients included with different type of urachal anomalies.

Urachal Anomalies	Urinary Symptoms	Cats	Dogs
*Intramural vesicourachal diverticulum*	Hematuria Pollakiuria Stranguria Urinary incontinence Asymptomatic	5/45 8/45 13/45 0 17/45	3/28 5/28 6/28 3/28 15/28
*Extramural vesicourachal diverticulum*	Hematuria Pollakiuria Stranguria Urinary incontinence Asymptomatic	4/12 2/12 4/12 0 5/12	1/7 1/7 0 0 5/7
*Urachal cysts*	Hematuria Pollakiuria Stranguria Urinary incontinence Asymptomatic	0 1/2 0 0 1/2	0 1/3 0 0 2/3
*Patent urachus*	Hematuria Pollakiuria Stranguria Urinary incontinence Asymptomatic	0 1/1 0 0 0	0 0 0 0 0
